# Tracking a trajectory of a moving stimulus by spike timing dependent plasticity

**DOI:** 10.1186/1471-2202-13-S1-P137

**Published:** 2012-07-16

**Authors:** Kazuhisa Fujita

**Affiliations:** 1 Department of Computer and Information Engineering, Tsuyama National Collage of Technology, Japan; 2 Department of Engineering Science, University of Electro-Communications, Japan

## 

The present study, we propose the recurrent network with spike timing dependent plasticity (STDP) that can track a trajectory of a moving stimulus. In STDP, synaptic efficiency is changed depending on the difference of firing time between pre- and postsynaptic neurons. There are many researches about temporal information processing. In the previous study, we have shown that the recurrent network with STDP can provide spatial filtering stimulating a static input [1]. Here we demonstrate the recurrent network with STDP can store spatiotemporal information of a stimulus.

The structure of the proposed network is 2-dimension array. A neuron in the network connects with the neighbor neurons through synapses whose changes are subjected to STDP. The learning window of STDP is applied to that found in hippocampus. We used STDP model proposed by Song and Abbott [2].

**Figure 1:**
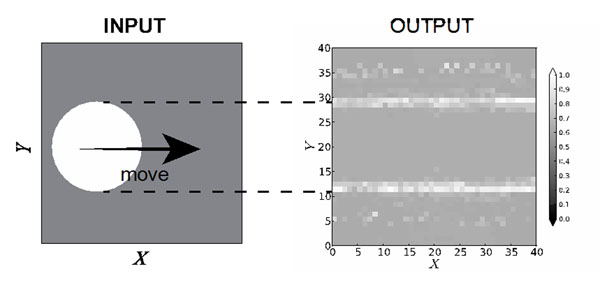
The input with a moving circle and the output of the network. The left figure is schematic image of the input. The right figure indicates the firing rate of the network.

The left figure in Figure 1 shows a schematic image of the input. The 2-dimensional input of the network consisted of a circle that moved straightly form left to right side. The white circle in the left figure indicates stronger inputs than the others. The diameter and the center of the circle is 20 and Y = 20, respectively. The right figure in Fig. 1 indicates peri-stimulus time histogram (PSTH) of the network. Two white lines appeared on PSTH. The lines indicate higher firing rates of the neurons than those of the others. The lines correspond to edges of the moving circle (see dashed line in Fig. 1.). The lines that represented higher firing rate could be considered as the trajectory of the moving circle of the input. This result suggests that the trajectory of the moving circle is accumulated in the synaptic weights of the neurons in the network. In the other words, the network could provide storage of a trajectory of a stimulus.

In the summary, the present study showed that the recurrent network with STDP could store a trajectory of a moving stimulus. STDP is also found in the midbrain of an electric fish. The electric fish can detect features of a moving object using electrosensory system. The object features present on the fish body surface as an electric image [3]. The function of the network shown in the present study helps explain how the electric fish can extract information from the features of an electric image generated by a moving object.
